# The effect of stress and anxiety associated with maternal prenatal diagnosis on feto-maternal attachment

**DOI:** 10.1186/1472-6874-11-33

**Published:** 2011-07-12

**Authors:** Sara J Allison, Julie Stafford, Dilly OC Anumba

**Affiliations:** 1 Academic Unit of Reproductive and Developmental Medicine, University of Sheffield, 4th Floor, Jessop Wing, Tree Root Walk, Sheffield, S10 2SF, UK; 2 Academic Unit of Reproductive and Developmental Medicine, University of Sheffield, South Yorkshire, UK; 3 Academic Unit of Reproductive and Developmental Medicine, University of Sheffield, South Yorkshire, UK

## Abstract

**Background:**

A couple's decision to undergo an invasive test based on a screening test result is a process associated with anxiety. The aim of this study was to determine whether anxiety and prenatal attachment were affected by undergoing an invasive test compared to women in early pregnancy and after a reassuring anomaly scan.

**Methods:**

200 women were recruited at booking, 14 women and 20 partners after an invasive test and 81 women following an anomaly scan. A questionnaire was completed using the Beck Anxiety Inventory and Maternal or Paternal Antenatal Attachment Scales.

**Results:**

Women who have had an invasive test have higher levels of anxiety compared to women at booking (P < 0.01) and after an anomaly scan (P = 0.002). Anxiety declines from booking to the time of an anomaly scan (P = 0.025), whilst attachment increases (P < 0.001). There is a positive correlation between anxiety and attachment in women who have had an invasive test (r = 0.479). Partners of women undergoing an invasive test experience lower levels of anxiety (P < 0.05).

**Conclusions:**

Women undergoing prenatal diagnostic procedures experience more psychological distress, which may be currently underestimated. Establishment of interdisciplinary treatment settings where access to psychological support is facilitated may be beneficial.

## Background

Prenatal screening for chromosomal abnormalities such as Down's syndrome is offered to all pregnant women in the United Kingdom as part of routine antenatal care. This initial screening process provides the mother-to-be and her partner with an estimation of the risk that their child may have an abnormality. The decision to undergo further more invasive diagnostic tests based on this probability then rests with the couple. The process of making this decision is associated with increased anxiety and stress for the pregnant woman and her partner.

The levels of stress and anxiety may be influenced by the information given, the counselling and psychological support provided, the results of the prenatal tests, and the outcome of the pregnancy. Stress and anxiety may also affect maternal feelings of attachment towards the fetus, and it is plausible that appropriate care and support may positively influence feto-maternal attachment by minimising stress.

### Anxiety in Pregnancy

Anxiety is defined as the psychological consequence of exposure to a real or imagined stress [1]. The decision to undergo a prenatal invasive test is undoubtedly associated with anxiety. There is much debate surrounding prenatal invasive testing as a means of reassurance for women identified as being at risk of carrying a fetus affected by a chromosomal or structural abnormality. While undergoing prenatal screening for genetic abnormalities may protect women from high levels of anxiety [2,3], it may also encourage women to focus on what may be wrong with the child, increasing levels of anxiety [4].

Previous research has clearly shown a relationship between undergoing an invasive test and increased anxiety levels [2,5,6] although receiving a normal result may in fact reduce the anxiety experienced later in the pregnancy [3].

Studies of prenatal anxiety and stress are important because there is increasing evidence that prenatal anxiety and stress may have long-term sequelae for both the pregnant woman and her fetus [7-9]. The mechanism by which these adverse effects occur is poorly understood but animal studies have shown that chronic stress may downregulate the fetal cortisol barrier enzyme response resulting in enhanced exposure of the fetus to maternal cortisol levels [10]. Abnormally high placental corticotropin releasing hormone (CRH) levels can cause vasodilatation resulting in reduced oxygen and nutrient delivery to the fetus [11]. Prolonged suboptimal conditions may lead to a state of 'thrifty' metabolism [12] predisposing to type II diabetes and obesity in later life [13]. If this physiological fetal programming occurs at a stage when the fetus is particularly sensitive to stressors there may be long-lasting impact upon memory, learning, affect and even frontal lobe executive function [9]. A study by Uno et al found that animals treated with synthetic glucocorticoids prenatally were shown to have a 30% reduction in hippocampal size [14].

Furthermore, stimulation of the autonomic nervous system (ANS) by anxiety or stress may lead to increased release of catecholamines such as noradrenaline, increasing uterine artery resistance and arterial pressure, causing a decrease in uterine blood flow and thus oxygen delivery to the fetus. One study found that high noradrenaline levels in pregnancy were negatively correlated to fetal head and abdominal circumferences [15].

### Prenatal Attachment

Any doubts raised concerning the health of the fetus that may lead to increased levels of stress and anxiety, such as a positive screening result or prenatal invasive test, may also interrupt the normal development of the psychological bond between the other and her fetus, which could lead to abuse, neglect and the ill-health of the child.

Feto-maternal attachment was described by Muller as 'the unique and affectionate antenatal relationship that develops between a mother and fetus' and it is characterised by the behaviours, attitudes, thoughts and feelings that demonstrate care and commitment to the fetus [16,17]. There is little previous research into prenatal attachment in women undergoing screening or an invasive diagnostic test in pregnancy. One study, using the Pregnancy Involvement List (PIL) to measure attachment, found that offering screening to women temporarily increases attachment [18] but another showed that undergoing a prenatal invasive test may decrease prenatal attachment, assessed using the Prenatal Attachment Interview (PAI) [19].

Two small studies have suggested that men have lower anxiety levels than their partners at the time of a prenatal invasive test [20,21] but did not report on paternal attachment.

### Purpose of the Study

This study aims to compare the relationship between anxiety and attachment in women and their partners undergoing an invasive test to women at an early gestation (who have not yet had any screening) and women at about 20 weeks gestation whose fetal morphology scan has shown no apparent abnormalities.

## Methods

Pregnant women and their partners attending the Jessop Wing, Royal Hallamshire Hospital, Sheffield were asked to participate between November 2009 and May 2010.

### Ethical Approval

The design of the study was given a favourable ethical opinion by the South Yorkshire Research Ethics Committee (reference number 09/H1310/64). Patients and their partners were asked to sign three copies of a consent form - one to be kept in the patients' medical records, one for the patient or partner to keep for reference and one for the researcher.

### Subjects

Three groups of pregnant women were recruited into the study along with one group of partners. Participants were excluded from the study if they had a current or past history of depression, anxiety or a significant medical condition that requires medication. Women with vaginal bleeding in the past two weeks or women who had suffered a previous miscarriage or had a family history of genetic disorder were also excluded.

The Q1 group were women recruited at booking - up to 18 weeks gestation - before they had any screening for Down's syndrome. Of 307 women approached, 107 were excluded whilst 200 women were recruited.

The Q2 group consisted of women who attended the feto-maternal unit and opted for an invasive genetic test following a screen-positive prenatal test for Down's syndrome or at the request of the pregnant woman due to maternal age. These women were between 12 and 18 weeks in gestation. Men who accompanied their spouses to this appointment were also recruited. Of 30 women and 24 partners who agreed to participate, 16 women and 4 men were excluded. Hence 14 women and 20 partners were included in the invasive test group. In total 11 couples were included.

The Q3 'anomaly scan' group consisted of women who were screen-negative for Down's syndrome and were recruited following a routine detailed scan at 18 to 22 weeks gestation that identified no major fetal abnormalities. 101 women were recruited in this group, of which 20 were excluded. Hence 81 women were included in the anomaly scan group.

### Measures

Anxiety and stress were assessed using the Beck Anxiety Inventory (BAI) [22]. The inventory consists of 21 items descriptive of subjective, somatic, or panic-related symptoms of anxiety. Self-reported answers are based on a 4-point Likert scale ranging from responses of 'not at all' to 'severe' in terms of the experience of that symptom over the past month. A high total score indicates more severe levels of anxiety.

Attachment was measured using the Maternal Antenatal Attachment Scale (MAAS) [23]. The scale consists of two factors - 'quality of attachment' which represents the quality of a mother's affective experiences such as closeness and tenderness; and 'time spent in attachment mode' which represents the intensity of preoccupation a mother experiences assessed through 19 items based on the feelings, behaviours and attitudes towards the fetus. The items are rated on a 5-point Likert scale. The total attachment score is computed by combining the two subscales. High scores reflect a positive quality of attachment. The paternal version of MAAS - the Paternal Antenatal Attachment Scale (PAAS) - consists of 16 items.

### Survey Instruments

Each participant was administered a survey instrument that included basic demographic information such as age, ethnicity, annual income, obstetric history, marital status and education; a scale for the assessment of anxiety and a scale for the assessment of prenatal attachment. They were asked to either complete the questionnaire while they waited or to post it back using the freepost envelope provided. Addtional file 1 gives an example of one of the questionnaires.

### Analyses

Data was entered into and analysed using SPSS 16.0 (Statistical Package for Social Science, Inc., Chicago, IL) for Windows. We compared maternal attachment and anxiety scales between the three groups using descriptive statistics, analysis of variance, T-tests and Mann-Whitney U tests as were appropriate.

We also employed multiple regression analysis, adjusting for the potential confounding effects of maternal age and parity on anxiety and attachment scores. Paternal data was described and anxiety compared with women in the invasive test group.

## Results

### Demographics

Table 1 shows the demographics of the women included in the booking (Q1), invasive test (Q2) and anomaly scan (Q3) groups.

**Table 1:** clinical characteristics of women at booking, undergoing an invasive test and after an anomaly scan.

	Booking Group (Q1) n = 200	Invasive Test Group (Q2) n = 14	Anomaly Scan Group (Q3) n = 81
Maternal age (years)	30.4 (5.1)	36.7 (5.8)	30.0 (5.5)
Gestation (weeks)	12 [8-18]	13.5 [11-21]	20 [18-27]
Gravidity	1 [1-7]	1.5 [1-6)	1 [0-7]
Parity	0 [0-5]	0 [0-5]	0 [0-4]
Ethnicity: White British	81.5%	78.6%	77.8%
Education: Further	76.0%	71.4%	80.2%
Marital Status: Married	60.0%	14.3%	66.7%
Employed: Yes	78.5%	71.4%	76.5%

Data are shown as mean (SD) or median [range].

Participants in Q2 were older than women in the Q1 and Q3 (P < 0.001). 15 women in Q1 (7.5%), 1 woman in Q2 (7.1%) and 6 women in Q3 (7.4%) were current smokers with a range of 1 - 20 cigarettes smoked a day.

### Anxiety

**Figure 1:**
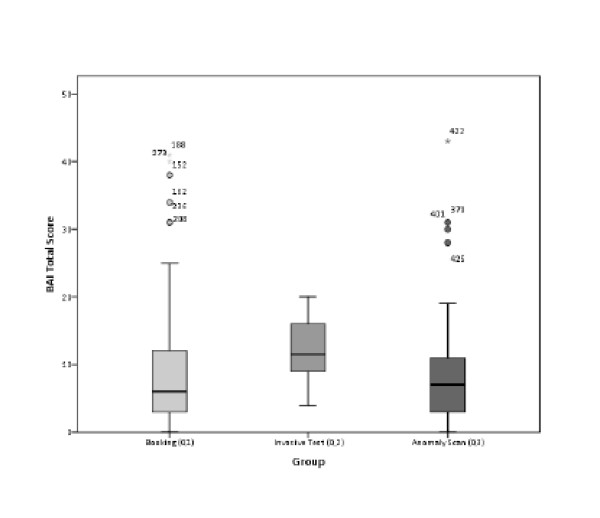
**Distribution of total anxiety scores between groups; the mean anxiety score is higher for Q2 than the other two cohorts**.

The mean total score on the BAI was 8.34 for Q1 (SD: 7.47) ranging from 0 - 41; 12.36 for Q2 (SD: 4.47) ranging from 4 - 20; and 8.19 for Q3 (SD: 7.43) ranging from 0 - 43. The invasive test group (Q2) had significantly higher levels of anxiety compared to the booking (P = 0.003) and anomaly scan groups (P = 0.002). This can be shown graphically in Figure 1f.

If we classify levels of anxiety, a score of 0-7 indicates minimal anxiety; 8-15 indicates mild anxiety; 16-25 indicates moderate anxiety and a score of 26 and above indicates severe anxiety [22]. Women in Q2 experienced more severe anxiety, albeit within the 'minimal' range, on average (mean: 2.29; SD: 0.79 vs. mean: 1.56; SD: 0.61 for Q1 and mean: 1.54; SD: 0.82 for Q3) but with 35.7% (n = 5) experiencing moderate anxiety.

### Attachment

Table 2 shows the mean total MAAS scores by group. Higher levels of attachment were experienced by women of increasing gestation (Q1 vs. Q3 P < 0.001). Using the cut-off scores employed by Pollock and Percy in previous studies, attachment assessed by MAAS may be categorised into 'high' (a score of seventy-six and above) and 'low' [24,25]. Given these categories, the further along a woman was in her pregnancy, the 'higher' her attachment was. 49% of women in Q1 had 'high' attachment compared to 64.2% of women in Q3 (P = 0.021).

**Table 2:** total BAI and MAAS scores by group.

	Booking Group (Q1) n = 200	Invasive Test Group (Q2) n = 14	Anomaly Scan Group (Q3) n = 81
Total BAI score	8.3 (7.5)	12.4 (4.5)	8.2 (7.4)
Total MAAS score	74.7 (7.1)	75.2 (7.1)	78.5 (7.1)

Data are shown as mean (SD).

Looking independently at the subscales of MAAS, 16.5% of women in Q1, 14.3% of women in Q2 and 33.3% of women in Q3 had a 'positive' quality of attachment (scoring 49 or above) suggesting that quality of attachment increases throughout pregnancy (Q1 vs. Q3 P = 0.002).

In terms of intensity of preoccupation with their babies, 42.5% of women in Q1, 57.1% of women in Q2 and 53.1% of women in Q3 were 'preoccupied' (scoring 27 or above) rather than 'disinterested'.

**Figure 2:**
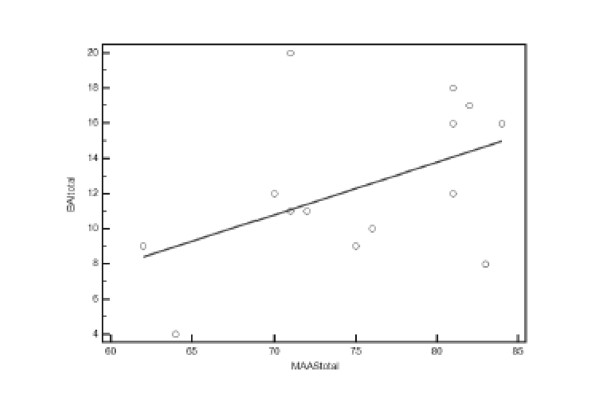
**Correlation between total BAI and MAAS scores in Q2; there is moderate positive correlation between the two variables**.

Figure 2 shows that moderate positive correlation was found between anxiety and prenatal attachment in women in the invasive test group (r = 0.479, n = 14, P = 0.083) with the variables sharing 23% of their variance. No such correlation was demonstrated for women in Q1 and Q3.

Table 3 shows the anxiety scores for women in each group by attachment subscales. Women who had a 'negative' quality of attachment and were more 'preoccupied' with their pregnancy had higher levels of anxiety. There was a statistically significant difference in the proportion of women in Q2 who had 'high' attachment experiencing higher levels of anxiety (mean: 13.86; SD: 3.85) compared to Q1 (mean: 9.11; SD: 7.97; P = 0.015) and Q3 (mean: 8.48; SD: 7.35; P = 0.004). Women in Q2 who had a 'negative' quality of attachment or were 'preoccupied' with their pregnancy were also more likely to experience higher levels of anxiety than any other group (P < 0.05).

**Table 3:** mean total BAI scores for MAAS attachment subscales by group.

		Group Total BAI Scores		
		**Booking Group** **(Q1) n = 200**	**Invasive Test** **Group (Q2)** **n = 14**	**Anomaly Scan** **Group (Q3)** **n = 81**

Maternal total score (global attachment)	Low	7.6 (6.9)	10.9 (4.8)	7.7 (7.7)
	
	High	9.1 (8.0)	13.9 (3.9)	8.5 (7.4)

Quality of attachment	Negative	9.0 (7.7)	11.9 (4.2)	8.8 (6.8)
	
	Positive	5.0 (4.9)	15.0 (7.1)	6.9 (8.6)

Intensity of Preoccupation	Disinterested	7.0 (6.0)	11.0 (5.2)	6.9 (6.1)
	
	Preoccupied	10.2 (8.8)	13.4 (3.9)	9.4 (8.3)

Data are shown as mean (SD).

Eight women were recruited on more than one occasion (into two groups of the study) allowing for longitudinal analyses. For the five women recruited into Q1 and Q3, anxiety decreased over time (P = 0.025) and attachment increased (P < 0.001). For the three women recruited into Q1 and Q2, anxiety levels appeared to dramatically increase after undergoing an invasive test and attachment levels appeared to fall but due to the small sample size these results were not statistically significant.

### Paternal Data

The mean total BAI score for partners included in Q2 was 8.20 (SD: 7.13), with scores ranging from 0 - 27. This is less than women included in this group suggesting that men experience lower levels of anxiety than their partners who have recently undergone an invasive test (P = 0.036).

The mean total PAAS score for partners included in Q2 was 54.05 (SD: 9.34), with scores ranging from 35 - 68. This represents mean % scores of the total maximum scores possible of 68.1% (54.1/80) compared to pregnant women scores of 79.2% (75.2/95).

For the 11 couples included in Q2, pregnant women experienced considerably higher levels of anxiety than their partners (mean: 13.55; SD: 3.93 vs. mean: 6.36; SD: 8.06; P = 0.015).

## Discussion

This study shows that women who have recently undergone an invasive test have higher levels of anxiety compared to women at earlier gestations first attending for antenatal care, and to women at a later gestation in mid-trimester immediately following a reassuring fetal morphology scan. This is consistent with findings from previous studies [5,6,26,27]. In a study with a longitudinal design Marteau et al found that anxiety levels in this group of women later fell to lower levels compared to other women, illustrating the long-term reassurance such a procedure may offer. This observation is likely to represent a normal response to the stress of making a difficult and risky decision and may aid effective decision-making.

Maternal stress has been shown to have severe effects on fetal brain development. Prenatal stress exposure during the development of the hippocampus, the component of the brain responsible for spatial memory tasks and associative and procedural memory, has been shown to decrease formation of synapses by at least 30% [28]. Offspring exposed to prenatal stress also show decreased numbers of glucocorticoid messenger ribonucleic acid (mRNA) and increased levels of corticotropin-releasing hormone (CRH) expression in the amygdala [7,8]. These changes may lead to a predisposition to mental health disorders such as depression as well as an increased sensitivity to stressful situation with a prolonged recovery and difficulties regulating emotions [9]. Indeed Van der Bergh found that prenatal anxiety in the third trimester of pregnancy was positively correlated with a difficult temperament in infants at 10 weeks and 7 months of age [29].

Prenatal stress has also been shown to affect behavioural development, thymic function and immune function in animal studies. Rhesus monkeys that were prenatally stressed have been shown to have lower amounts of exploratory behaviour and significantly more disturbance behaviour compared to controls [30]. Rats subjected to synthetic glucocorticoids prenatally showed a decrease in number of thymic T cells postnatal [31].

The effect of stress and its' associated immune changes in pregnancy may be a major factor in preterm labour. Two hypotheses have been put forward - firstly, that preterm birth may occur due to the significant immunological changes that occur as a result of stress without subsequent infection [32] and secondly, that preterm birth occurs through increased susceptibility to infections [33].

Stress has also been shown to decrease levels of the pregnancy supporting hormone progesterone and progesterone induces blocking factor (PIBF) which up regulates the production of Th1 cytokines [13,34]. This leads to increased prostaglandin production and increased uterine contractility and may cause pre-term labour [34].

Infection and inflammation account for up to 30% of preterm deliveries [35] and bacterial vaginosis, the most commonly associated infection with pre-term delivery, is more prevalent in pregnant women undergoing greater psychological stress [36].

These findings highlight the additional complications a woman and her unborn child may be at risk of as a result of the increased levels of anxiety associated with undergoing an invasive genetic test in pregnancy our observations have shown.

There is an increase in the total fetal attachment score from a pregnant women's booking visit to her fetal morphology scan in mid-trimester which many studies have also reported [24,37,38]. At variance with our observations, Lawson et al reported that women who opted for an invasive test had higher levels of attachment than women at screening or who had no test [19]. However our invasive test group demonstrated moderate positive correlation between anxiety and attachment, suggesting that heightened anxiety at the time of an invasive test may be a cause or consequence of stronger feto-maternal attachment. This is at variance with some previous studies that suggest that anxiety leads to a decrease in the quality of attachment, and that a lack of attachment may lead to increased anxiety [24,39-41]. Our observation supports the hypothesis that women who develop an emotional attachment to the fetus are more likely to experience anxiety about the pregnancy and unborn child [42,43] and that women who experience low levels of attachment are likely to feel less anxious about their pregnancy.

Partners have been shown to experience less anxiety than their respective partners, supporting previous research [20,21]. The difference in anxiety between women and their partners may be explained by the simple actuality that it is the pregnant woman who is carrying the baby and undergoes any invasive procedure rather than her partner and is therefore likely to have additional anxiety related to the pain and possible complications of that procedure.

### Study Limitations and Future Work

Considering the potential long-term benefits of undergoing an invasive test in terms of the reduction in anxiety levels it would be worthwhile undertaking longitudinal studies of women who have undergone invasive testing throughout pregnancy and into the postnatal period.

Most previous studies used the Spielberger State-Trait Anxiety Inventory (STAI) or the Pregnancy-Related Anxiety Questionnaire (PRAQ-r) to assess anxiety. The BAI is a widely employed survey instrument that focuses most on somatic symptoms in order to avoid correlation with depression as well as anxiety [44]. A study utilising both the BAI and STAI in adult psychiatric outpatients found that both scales demonstrated high internal reliabilities but that the BAI proved more useful as a screening instrument for a current anxiety disorder than the STAI [45,46]. However the BAI gives no indication of a woman's underlying 'trait' anxiety. Kowalcek et al found that women who had an increased trait anxiety score were more likely to have an increased state anxiety score after an invasive test [47]. The recently developed Beck Anxiety Inventory-Trait (BAIT) may be a useful survey instrument for future research [48]. Although we have not determined trait anxiety in our cohort of pregnant women we have tried to minimise the confounding influence of this on our observation by excluding women with previous histories of anxiety and depression.

Our pilot data on paternal anxiety and attachment in relation to invasive prenatal diagnosis should inform larger sufficiently powered studies aimed at elucidating the complex factors that are likely to influence feto-paternal attachment. Furthermore future work exploring the potential impact of clinical care interventions on maternal anxiety and fetal attachment are needed to optimise care for this client group.

## Conclusion

Women undergoing prenatal diagnostic procedures will experience more psychological distress and anxiety compared to women who are not at an increased risk of carrying a fetus affected by a chromosomal abnormality. Increased anxiety levels may in turn impact upon the extent to which a women and her partner can start to form a bond with their unborn child. It is therefore important to identify anxiety in pregnancy in order to offer each woman and her partner the most favourable environment and opportunities for a healthy pregnancy and relationship with their child.

The establishment of a multidisciplinary treatment situation, in which access to psychological support is offered, may be extremely beneficial for these women who are at an increased risk of experiencing anxiety.

## Competing interests

The authors declare that they have no competing interests.

## Authors' contributions

SJA participated in the study design, recruited patients into the study, performed the statistical analysis and drafted the manuscript. JS helped to recruit patients into the study. DOCA conceived the study, participated in its design and coordination and performed some of the statistical analysis. All authors read and approved the final manuscript.

## Author's Information

SJA conducted this research as part of her Bachelor of Medical Sciences Honours degree under the supervision of DOCA and with the help of JS.

## Pre-publication history

The pre-publication history for this paper can be accessed here:

http://www.biomedcentral.com/1472-6874/11/33/prepub

## Supplementary Material

Additional File 1 **Questionnaire for Booking Group (Q1)**. A questionnaire for women recruited at booking following a dating scan. The questionnaire includes sections on demographics, a scale to assess anxiety (BAI) and a scale to assess attachment (MAAS). Click here for file
