# Supplementary material for: The effect of stress and anxiety associated with maternal prenatal diagnosis on feto-maternal attachment
Source: BMC Womens Health. 2011 Jul 12;11:33. doi: 10.1186/1472-6874-11-33 (PMC3148201; doi:10.1186/1472-6874-11-33)
Supplement: Additional File 1 — Questionnaire for Booking Group (Q1). A questionnaire for women recruited at booking following a dating scan. The questionnaire includes sections on demographics, a scale to assess anxiety (BAI) and a scale to assess attachment (MAAS). [file 1472-6874-11-33-S1.DOCX]

[
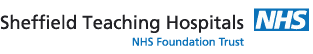
](http://www.sth.nhs.uk/index.php)

**G1 QUESTIONNAIRE (booking visit)**

Please take your time to fill in this questionnaire and hand it to one of our research team or place in the box provided. It should take approximately 15 minutes.

If you feel you need more time to decide whether or not you wish to take part in this study you may take it home and fill it in at your leisure before returning it in the stamp-addressed envelope provided.

All details will be kept confidential and anonymised.

**Patient Details**

Hospital Number (if known): ____________________

Title (please delete as appropriate): Miss/Ms/Mrs/Mr Other (please specify): _________

Surname: ____________________________________

First Name: __________________________________

Address: ____________________________________

____________________________________

Post Code: ____________________________

Contact Number: ______________________________

**Demographics**

Date of Birth: ___/___/___

Gender (please tick the box which applies to you): M F

Race/Ethnicity: White British

Irish

Black

Asian

Chinese

Mixed

Are you currently employed? Yes - Occupation: _________________________ No

Annual Income: Under £5,000

£5,000-£10,000

£10,000-15,000

£15,000-£20,000

£20,000-£25,000

£25,000-£30,000

£30,000-£40,000

£40,000-£50,000

£50,000-£75,000

£75,000-£100,000

More than £100,000

Education: Primary School

Secondary School

Further Education e.g. university, college

Marital Status: Single Married Divorced Separated Widowed

Smoking Status: Smoker – How many cigarettes do you smoke a day? _______

Non-smoker

**Medical History**

How many weeks pregnant are you? _____

Have you been pregnant before? Yes No

If yes, how many previous pregnancies have you had? ___

How many live children were born? ___

Have you ever had a miscarriage? Yes No

Do you have a family history of genetic disorders? Yes, please give details __________

____________________________

No

Have you had any vaginal bleeding in the last two weeks? Yes No

Have you had, or currently have, any of the following conditions (please tick all boxes that apply)?

| Heart disease  Stroke  Diabetes  Epilepsy  Thyroid disease  Emphysema or chronic bronchitis  High blood pressure  Asthma  Substance Misuse | Cancer  Splenectomy  Kidney disease  Schizophrenia  Depression  Bipolar affective disorder  Anxiety disorder Sub-fertility |
| --- | --- |

Please give details of any medications you are taking: _______________________________

___________________________________________________________________________

**Anxiety**

Below is a list of common symptoms of anxiety.

Please read each item in the list carefully and indicate how much you have been bothered by that symptom during the **past month**, including today.

Please circle the number in the column next to the symptom that best applies to how you feel.

| **Symptom** | **Not at all** | **Mildly** – it didn’t bother me much | **Moderately** – it wasn’t pleasant at times | **Severely** – it bothered me a lot |
| --- | --- | --- | --- | --- |
| Numbness or tingling | 0 | 1 | 2 | 3 |
| Feeling hot | 0 | 1 | 2 | 3 |
| Wobbliness in legs | 0 | 1 | 2 | 3 |
| Fear of the worst happening | 0 | 1 | 2 | 3 |
| Unable to relax | 0 | 1 | 2 | 3 |
| Dizzy or lightheaded | 0 | 1 | 2 | 3 |
| Heart pounding or racing | 0 | 1 | 2 | 3 |
| Unsteady | 0 | 1 | 2 | 3 |
| Terrified or afraid | 0 | 1 | 2 | 3 |
| Nervous | 0 | 1 | 2 | 3 |
| Feeling of choking | 0 | 1 | 2 | 3 |
| Hands trembling | 0 | 1 | 2 | 3 |
| Shaky | 0 | 1 | 2 | 3 |
| Fear of losing control | 0 | 1 | 2 | 3 |
| Difficulty in breathing | 0 | 1 | 2 | 3 |
| Fear of dying | 0 | 1 | 2 | 3 |
| Scared | 0 | 1 | 2 | 3 |
| Indigestion | 0 | 1 | 2 | 3 |
| Faint | 0 | 1 | 2 | 3 |
| Face flushed | 0 | 1 | 2 | 3 |
| Hot or cold sweats | 0 | 1 | 2 | 3 |

**Attachment**

These questions are about your thoughts and feelings about the developing baby over the **past** **two weeks**. Please tick one box only in answer to each question.

1. Over the past two weeks I have thought about, or been preoccupied with the baby inside me:

|  | Almost all the time |
| --- | --- |

|  | Very frequently |
| --- | --- |

|  | Frequently |
| --- | --- |

|  | Occasionally |
| --- | --- |

|  | Not at all |
| --- | --- |

1. Over the past two weeks when I have spoken about, or thought about the baby inside me I got emotional feelings which were:

|  | Very weak or non-existent |
| --- | --- |

|  | Fairly weak |
| --- | --- |

|  | In between strong and weak |
| --- | --- |

|  | Fairly strong |
| --- | --- |

|  | Very strong |
| --- | --- |

1. Over the past two weeks my feelings about the baby inside me have been:

|  | Very positive |
| --- | --- |

|  | Mainly positive |
| --- | --- |

|  | Mixed positive and negative |
| --- | --- |

|  | Mainly negative |
| --- | --- |

|  | Very negative |
| --- | --- |

1. Over the past two weeks I have had the desire to read about or get information about the developing baby. This desire is:

|  | Very weak or non-existent |
| --- | --- |

|  | Fairly weak |
| --- | --- |

|  | Neither strong nor weak |
| --- | --- |

|  | Moderately strong |
| --- | --- |

|  | Very strong |
| --- | --- |

1. Over the past two weeks I have been trying to picture in my mind what the developing baby actually looks like in my womb:

|  | Almost all the time |
| --- | --- |

|  | Very frequently |
| --- | --- |

|  | Frequently |
| --- | --- |

|  | Occasionally |
| --- | --- |

|  | Not at all |
| --- | --- |

1. Over the past two weeks I think of the developing baby mostly as:

|  | A real little person with special characteristics |
| --- | --- |

|  | A baby like any other baby |
| --- | --- |

|  | A human being |
| --- | --- |

|  | A living thing |
| --- | --- |

|  | A thing not yet really alive |
| --- | --- |

1. Over the past two weeks I have felt that the baby inside me is dependent on me for its well-being:

|  | Totally |
| --- | --- |

|  | A great deal |
| --- | --- |

|  | Moderately |
| --- | --- |

|  | Slightly |
| --- | --- |

|  | Not at all |
| --- | --- |

1. Over the past two weeks I have found myself talking to my baby when I am alone:

|  | Not at all |
| --- | --- |

|  | Occasionally |
| --- | --- |

|  | Frequently |
| --- | --- |

|  | Very frequently |
| --- | --- |

|  | Almost all the time I am alone |
| --- | --- |

1. Over the past two weeks when I think about (or talk to) my baby inside me, my thoughts:

|  | Are always tender and loving |
| --- | --- |

|  | Are mostly tender and loving |
| --- | --- |

|  | Are a mixture of both tenderness and irritation |
| --- | --- |

|  | Contain a fair bit of irritation |
| --- | --- |

|  | Contain a lot of irritation |
| --- | --- |

1. The picture in my mind of what the baby at this stage actually looks like inside the womb is:

|  | Very clear |
| --- | --- |

|  | Fairly clear |
| --- | --- |

|  | Fairly vague |
| --- | --- |

|  | Very vague |
| --- | --- |

|  | I have no idea at all |
| --- | --- |

1. Over the past two weeks when I think about the baby inside me I get feelings which are:

|  | Very sad |
| --- | --- |

|  | Moderately sad |
| --- | --- |

|  | A mixture of happiness and sadness |
| --- | --- |

|  | Moderately happy |
| --- | --- |

|  | Very happy |
| --- | --- |

1. Some pregnant women sometimes get so irritated by the baby inside them that they feel like they want to hurt it or punish it:

|  | I couldn’t imagine I would ever feel like this |
| --- | --- |

|  | I could imagine I might sometimes feel like this, but I never  actually have |
| --- | --- |
|  |  |

|  | I have felt like this once or twice myself |
| --- | --- |

|  | I have occasionally felt like this myself |
| --- | --- |

|  | I have often felt like this myself |
| --- | --- |

1. Over the past two weeks I have felt:

|  | Very emotionally distant from my baby |
| --- | --- |

|  | Moderately emotionally distant from my baby |
| --- | --- |

|  | Not particularly emotionally close to my baby |
| --- | --- |

|  | Moderately close emotionally to my baby |
| --- | --- |

|  | Very close emotionally to my baby |
| --- | --- |

1. Over the past two weeks I have taken care with what I eat to make sure the baby

gets a good diet:

|  | Not at all |
| --- | --- |

|  | Once or twice when I ate |
| --- | --- |

|  | Occasionally when I ate |
| --- | --- |

|  | Quite often when I ate |
| --- | --- |

|  | Every time I ate |
| --- | --- |

1. When I first see my baby after the birth I expect I will feel:

|  | Intense affection |
| --- | --- |

|  | Mostly affection |
| --- | --- |

|  | Dislike about one or two aspects of the baby |
| --- | --- |
|  |  |

|  | Dislike about quite a few aspects of the baby |
| --- | --- |

|  | Mostly dislike |
| --- | --- |

1. When my baby is born I would like to hold the baby:

|  | Immediately |
| --- | --- |

|  | After it has been wrapped in a blanket |
| --- | --- |

|  | After it has been washed |
| --- | --- |

|  | After a few hours for things to settle down |
| --- | --- |

|  | The next day |
| --- | --- |

1. Over the past two weeks I have had dreams about the pregnancy or baby:

|  | Not at all |
| --- | --- |

|  | Occasionally |
| --- | --- |

|  | Frequently |
| --- | --- |

|  | Very frequently |
| --- | --- |

|  | Almost every night |
| --- | --- |

1. Over the past two weeks I have found myself feeling, or rubbing with my hand, the outside of my stomach where the baby is:

|  | A lot of times each day |
| --- | --- |

|  | At least once per day |
| --- | --- |

|  | Occasionally |
| --- | --- |

|  | Once only |
| --- | --- |

|  | Not at all |
| --- | --- |

1. If the pregnancy was lost at this time (due to miscarriage or other accidental event) without any pain or injury to myself, I expect I would feel:

|  | Very pleased |
| --- | --- |

|  | Moderately pleased |
| --- | --- |

|  | Neutral (i.e. neither sad nor pleased; or mixed feelings) |
| --- | --- |

|  | Moderately sad |
| --- | --- |

|  | Very sad |
| --- | --- |
